# Energy Solutions, Neo-Liberalism, and Social Diversity in Toronto, Canada

**DOI:** 10.3390/ijerph8010185

**Published:** 2011-01-19

**Authors:** Cheryl Teelucksingh, Blake Poland

**Affiliations:** 1 Sociology Department, Ryerson University, 350 Victoria Street, Toronto, Ontario, M5B 2K3, Canada; 2 Dalla Lana School of Public Health, University of Toronto, 155 College Street, Toronto, Ontario, M5T 3M7, Canada ; E-Mail: blake.poland@utoronto.ca

**Keywords:** environmental justice, energy policy, Toronto, green capitalism

## Abstract

In response to the dominance of green capitalist discourses in Canada’s environmental movement, in this paper, we argue that strategies to improve energy policy must also provide mechanisms to address social conflicts and social disparities. Environmental justice is proposed as an alternative to mainstream environmentalism, one that seeks to address systemic social and spatial exclusion encountered by many racialized immigrants in Toronto as a result of neo-liberal and green capitalist municipal policy and that seeks to position marginalized communities as valued contributors to energy solutions. We examine Toronto-based municipal state initiatives aimed at reducing energy use while concurrently stimulating growth (specifically, green economy/green jobs and ‘smart growth’). By treating these as instruments of green capitalism, we illustrate the utility of environmental justice applied to energy-related problems and as a means to analyze stakeholders’ positions in the context of neo-liberalism and green capitalism, and as opening possibilities for resistance.

## 1. Introduction

While there are many streams of environmentalism in Canada, a discourse of green capitalism has increasingly dominated Canada’s environmental movement and is widely endorsed by policy makers [1]. Proponents of this view seek to add “environmentally friendly” values to the *status quo* liberal democratic ideals of Canadian society [2]. Green capitalism offers the appearance of a “win-win” scenario where environmental solutions are rendered compatible with a pro-growth, urban development orientation. However, two additional trends complicate the picture. First, environmental laws, health and safety legislation, consumer protection and other regulatory safeguards are often reduced by environmental policy makers in the interests of the perceived efficiency of the market [3]. Second, neo-liberal government cutbacks, a racially divided labour market, and urban renewal initiatives contribute to both the racialization of poverty [4] and spatial segregation [5] for many immigrants and members of racialized groups. In this paper, we advance environmental justice, as an alternative to mainstream environmentalism, that seeks to address systemic problems of social and spatial exclusion encountered by many racialized immigrants in Toronto, and to position marginalized communities as important contributors to energy solutions. Toronto-based initiatives to reduce energy use, while concurrently aimed at stimulating growth (specifically green economy/green jobs and ‘smart growth’) are examined as instruments of green capitalism that have the potential to threaten environmental justice and diversity. We also consider what an environmental justice frame suggests in terms of more equitable energy policy.

Most jurisdictions in Canada today—including Toronto—are facing an ‘energy contradiction’ [6]: high levels of energy use underwrite continued economic growth and urban expansion, that are increasingly acknowledged as unsustainable in light of: climate change and growing concerns about energy security; global oil supplies; and the environmental consequences of chasing after increasingly remote and ‘unconventional’ sources (e.g., the Alberta Tar Sands, BP Gulf of Mexico oil spill). High energy demand has translated into political (and economic) pressure to increase energy supplies so that current lifestyles can continue uninterrupted. This concurrent emphasis on expanding energy demand and energy supply, rests on an assumption that technological innovation, driven by market competition, can overcome energy challenges associated with rising energy costs, limits to fossil fuel supplies, aging energy infrastructure, including potential power blackouts [7].

While this paper focuses predominately on Toronto and its surrounding region, we submit that the interactions between unsustainable energy use, immigrant communities, and the ideologies of neo-liberalism and green capitalism offer considerable explanatory power in understanding energy policy in other urban contexts.

## 2. Environmental Justice

In this paper, we propose environmental justice as a framework to incorporate marginalized households and communities into a sustainable vision of Toronto. Environmental justice is a social movement and theoretical framework that seeks to merge issues of social justice into environmental movements. Environmental justice started in the 1980s in the United States as a response to the disproportionate burden borne by communities of colour [8,9]. Communities that were not previously involved in the mainstream environmental movement began to question inequitable distributions of environmental costs. By focusing on environmental problems and their link to social inequality, environmental justice addresses a wide variety of social and environmental problems (e.g., harmful practices in housing, and land use, as well as the need for better quality health care—[10]). The environmental justice movement has also identified issues of procedural (in)justice, such as the exclusion of marginalized communities from access to information, fair hearings, and equal participation in environmental, development, and land use matters that directly impact their quality of life [11].

As a critique of policy, environmental justice has been applied to identify what can be done to remedy as well as prevent environmental injustices. Connecting environmental justice theory and practice in the US has led to successes that have translated into policy gains and public recognition. As a milestone in the movement, in 1991, President Clinton signed Executive Order 12898 which reinforces the *1964 Civil Rights Act* and prohibits discriminatory practices in federally funded government programs that impact on the environment of minority and low income populations.

Environmental justice research and activism in Canada and in Toronto has emerged more slowly and without the same grassroots and political commitment that characterized the US movement [10,12,13]. According Gosine and Teelucksingh [10], the language and activism around environmental justice has not gained significant ground, in part, due to the lack of a US-style civil rights movement in Canada and collective denial of the ongoing legacy of colonialism and racial oppression that is still experienced on a daily basis by Aboriginal, immigrant, and other racialized communities in Canada. These theorists argue that the links between systemic forms of institutional racism; the environmental inequalities relating to natural resource access; and exposures to environmental risks are either overlooked or underestimated. Many experiences of environmental injustice, while not specifically named and labeled as such, have had an enormous impact on the lives of Aboriginal and racialized communities in Canada, and have contributed to these communities’ marginalized status vis-à-vis the Canadian state. Haluza-DeLay *et al.* [14] and Masuda *et al.* [15] suggest that research consistent with the objectives of environmental justice has had a long history in Canadian universities, government agencies, and non-governmental organizations, although this research may have fallen under different banners such as health, natural resource, and Aboriginal oriented research.

In this paper, we focus on racialized communities and immigrant populations. Racialization is a lens through which to examine dominant social relations and the ideology of multiculturalism as it manifests in Toronto. By considering racial meanings as part of an ongoing historical process, racialization is an interrelated component of other political, economic discourses, including both class and immigration status [16,17]. From an environmental justice perspective, treating oppressions as competing factors ignores the fact that both the social order and the capitalist system are dependent on the exploitation of multiple (indeed all) subordinate social relations and the environment.

Energy concerns—and the steps taken to alleviate them - translate into household level impacts on housing, transportation and subsistence, and these impacts are felt differentially by different segments of society. Starting in the 1990s, American environmental justice researchers and activists have reinforced the connection between energy use/depletion and social issues at the micro level. As Lee [18] argues, ‘poor people and people of colour are benefitting the least and paying the most for this society’s wasteful dependency on fossil fuels, and nuclear power, and from the resulting air pollution’ ([18], p. 1). Household energy consumption is tied to income; for instance, poor households cannot afford larger houses and/or multiple appliances like gas plasma TVs that consume more energy [18]. Despite using less energy, these households are more vulnerable: higher heating prices may contribute to some low-income households losing their homes altogether. Similarly, poor households are more likely to drive cars that are older and less energy efficient, and/or rely more heavily on public transportation [18].

There is also a procedural justice dimension to energy, as poor communities and racialized communities have been largely excluded from the arenas where energy policy decisions are made. Marginalized people often have less information about energy rebates, although their houses and apartments are among the least energy efficient, and the solutions that are presented (e.g., investing in home improvement measures such as insulation or Energy Star appliances) are often not feasible (e.g., for renters). More broadly, while the natural resources needed to produce energy are often located in or near poor or Aboriginal communities, the views of these communities are often not given much weight in decisions about the extraction and production of energy, despite the potential negative impact on the physical environment and communities nearby [19,20].

The connection between environmental justice and the study of environmental policy in Canada remains under-explored in scholarship in part due to the emphasis on applying environmental justice to explain problems of distributional injustices. In response to this identified gap, in this paper we apply environmental justice to examine the theory and practice of environmentalism and energy policy in Toronto.

## 3. Bringing Environmental Justice to Environmental Policy: A Critique of Neo-Liberalism and Green Capitalism

A political economy critique of capitalist accumulation and power relations is a defining feature of environmental justice research and practice [21,22]. This provides a structural framework for understanding the actions of powerful stakeholders who disproportionately control the “free” market. In addition, an environmental justice analysis unpacks the systemic nature of environmental and social inequalities emerging from neo-liberal policy regimes and the links to market driven privatization and green capitalism initiatives.

Manifestations of neo-liberalism globally and in Toronto include: restructuring of state policies to reduce ‘red tape’, oversight, and costs; government stated preference for self-regulation over ‘interference’ in the market; increased privatization; efforts to create a ‘favourable climate’ for foreign investors; and greater competitiveness. It should be noted that, increasingly, this ‘laissez-faire’ rhetoric belies and is coupled with heavy subsidies to oil extraction and other mega-projects, costly ‘bail-outs’ of financial sector players, increased military spending, and mounting government debt. Neo-liberal reforms (both the reductions in public services and programs and channeling of public money into military, financial, oil and gas and other ventures that disproportionately benefit select private players, reinforces the divide between those with power and resources and those without. From the perspective of achieving environmental justice and addressing social inequality, neo-liberalism reduces the ability of the state to solve social problems through welfare state type policies. According to Coburn [23], neo-liberal ideologies are based upon the belief that the market is the best (most practical, efficient, and, perhaps, even ethical) distributor of social and economic goods. It is worth noting that in Toronto, there have been many acts of resistance to state imposed neo-liberalism. These have ranged from protests and tent cities, advocating for alternative housing, education and food systems. These various forms of resistance seek both to destabilize dominant power relations and to highlight social inequalities. They are also, concurrently, a way to ultimately change dominant norms and values and to influence the formal processes of the state.

A view to applying environmental justice to a critique of dominant neo-liberal state policies must consider the manner in which neo-liberalism’s negative impact on social policy from the perspective of racialized people becomes layered onto environmental policy. In multicultural Toronto, economic, spatial, and social disparities are infused with racial meanings; even if the disparities are not directly motivated by race. Many theorists [4,24,25] have drawn attention to the implications of immigration flows from the global South and the resulting challenge that racial/ethnic diversity poses to social cohesion in Toronto. Starting in the 1980s, many new immigrants to Toronto experienced difficulties translating their levels of education and training into the desirable jobs in the Canadian labour market. These waves of immigrants from the global South often also encountered systemic forms of racism and exclusion. At the point when racialized new immigrants were most in need of assistance with their social and economic integration into Toronto, federal and provincial governments’ withdrew from supportive social programs in areas such as health care, education, and housing [25]. Neo-liberal social policies have strong similarities to the Ontario provincial government’s environment policy regimes that continue to reflect ideologies of neo-liberalism and green capitalism.

Winfield and Jenish [26] highlight important components of the Ontario Conservative government’s move toward imposing neo-liberal policies in Ontario starting in the late 1990s. According to Winfield and Jenish [26] and Winfield [27], in terms of the environment, neo-liberalism took the form of reduced opportunities for public participation in decision making; the repeal of land-use planning requirements intended to curb urban sprawl; and reduced budgets for provincial and local environmental and natural-resources agencies, including cuts in the funding and staffing for the Ontario Ministry of the Environment. Under neo-liberal regimes, the regulation and management of resources, such as energy need to conform to market conditions To the extent that these neo-liberal regimes’ intentions are also manifest in the energy sector, the result is reduced protection for individuals and households in a context where negative costs associated with energy use, such as increased greenhouse gases, inefficient use of energy, and associated health problems, may be escalating. Since the end of the Conservative regime in Toronto and the rise of the Ontario Liberals, this legacy persists in less overt forms of neo-liberalism.

Since the 1990s, the green capitalist approach to environmentalism has been taken up by municipal government officials, and urban planners [28] as a means to position growth-oriented urban development as compatible with the environment. Privatization has moved the objectives of urban development and the governance of particular utilities into the hands of private corporations, outside of the control and regulation of government. In this regard, Kipfer and Keil note that, ‘[I]t is essentially market efficiency and service delivery that dominate the discussion here above concerns of ecological sustainability, democracy, or social justice’ ([28], p. 141).

It is also worth emphasizing that there are different types of neo-liberalism and green capitalism. Holifield [29] argues that, under the US Clinton administration a version of neo-liberalism and environmental justice merged together. Holifield [29], drawing on Jessop [30], explains that “‘roll-out’ or ‘deep’ neo-liberalism of the 1990s [in the US] focused on constructing new institutions of federal intervention to consolidate and deepen neoliberal hegemony” ([29], p. 203). For example, environmental justice initiatives embedded in urban renewal projects under the Clinton administration co-opted the discourse of environmental justice, such as community empowerment, citizen involvement, and economic self-sufficiency, in a manner that was nevertheless (more) compatible with neo-liberalism. Similarly, neo-liberal policy initiatives in Toronto have moved to an emphasis on collaborations that seek to bring together public, private, ENGO, and community interests. As discussed below and as applied to Ontario’s *Green Energy Act* [31], these multi-stakeholder plans do not include the financial supports and accessibility that allow for marginalized stakeholders to benefit equally. Thus, it is only on the surface that government policies appear to address procedural inequalities. An environmental justice framework would point to ongoing social and environmental inequalities connected to policy outcomes.

For the balance of this paper, drawing on a review of the relevant grey literature, we apply environmental justice as a lens to analyze green economy/green jobs and smart growth policies and to suggest directions for a more just and sustainable energy policy. First, we start by providing the background context for Toronto in terms of its energy uses, Toronto’s orientation to being a global city, and its changing demographic patterns.

## 4. Toronto: Energy Use, Globalization, and the Dynamics of Immigrant Settlement

Natural resource wealth and energy resources, in particular, play an important role in the Canadian economy. In 2006, energy resources accounted for 57% of Canada’s total resource wealth [32]. Canada is a major supplier of energy to the US, including the exploitation of the Tar Sands in Alberta [33]. Energy wealth in Canada results in regional differences in terms of how energy conservation and sustainability are perceived and enacted. Regional tensions between large energy producers and energy consumers have been an integral part of the federal political landscape in Canada. According to Toronto’s Sustainable Energy Plan, ’Toronto’s energy mix is dominated by natural gas, accounting for 63% of all the energy used (except for transportation) in Toronto, while local renewable energy resources provide only 0.6%’ ([7], p. 4). From the perspective of the Toronto municipal government, energy expenditures represent lost revenue for the local economy and it makes Toronto businesses and households vulnerable to changing costs and supplies from external energy suppliers. Toronto’s total electricity use per capita is higher in comparison to other large urban centres, such as New York, Greater London, and Tokyo ([7], p. 4).

Recently, Toronto has experienced problems that have provided greater reasons to reconsider the sustainability of its reliance on distant energy sources. Toronto has been vulnerable to black outs, particularly in the high demand summer months. This concern is likely to be exacerbated as temperatures rise with global warming, putting further pressure on the peak summer-time demand for electricity. Also, ‘Toronto’s Medical Officer of Health estimates that 1,700 Torontonians died in 1999 due to air pollution. Much of this pollution comes from the way we use energy for transportation, in buildings, and in our infrastructure’ ([7], p. 38). Plans to promote local renewable energy sources must also take into account the ways in which potential environmental health issues vary across a city as diverse as Toronto.

It is important to consider Toronto’s energy concerns from the perspective of Toronto’s positioning as a ‘global city’ and how recent waves of immigrants have integrated here socially and spatially [34]. Toronto’s rising global status is characterized by three important political and economic processes: (1) the cities‘ enhanced role as a financial and service centre for the new global economy and the economic and spatial inequalities emerging from economic restructuring; (2) population growth and the increasing racialization of Toronto via new immigration, and; (3) stress on the housing market and on the municipal government’s ability to provide affordable housing for those in need [17].

As a global city, Toronto has become central in coordinating the international division of labour, which involves multinational corporations with various locations of production and distribution, and the global movement of financial capital. Since the 1980s, globalization and the move toward cleaner, technology-driven “new economy” sectors (including information technology, high finance, and new media) have reshaped the demographic geography of Toronto, as downtown residents have become increasingly white-collar. To wit, professional classes and groups with capital have been drawn to employment and housing in Toronto’s inner city [34–36]. Since the liberalization of Canadian federal immigration policy in the late 1960s, new waves of immigrants are integrated into a racialized division of labour between the highly skilled and highly paid professional strata (largely white), on the one hand, and the low-skilled and low-paid service sector strata (largely racialized), on the other [4]. Simultaneously, as described above, since the 1990s neo-liberal agendas have resulted in federal, provincial, and municipal government cut-backs to subsidized housing, health care, education, and the environment [1].

Recent immigrants have more dispersed settlement patterns, with more settling directly in the suburbs than in the city core [5,37]. The spatial segregation documented by Hulchanski *et al.* [5] shows fewer new immigrants and members of racialized groups finding housing in downtown Toronto, where the property values have increased and many new residential developments are geared to the affluent consumer. Young and Keil note that the four regional municipalities, which make up the suburban areas of the City of Toronto, ‘have population growth at four times [the rates of] [the rest] of the city’ ([1], p. 144). Much of the growth in these suburban areas is low density, consisting of “sprawling subdivisions” punctuated by pockets of older high-rise apartment blocks poorly served by surrounding services. Unequal access to downtown Toronto housing has taken on increasingly racialized dimensions, which impacts the social-spatial context of environmental issues in Toronto. Arguably, social tensions arising in part from the social-spatial organization of Toronto requires particular types of energy policy that are best suited to meeting the settlement, transportation, and consumer needs of all Torontonians, including the growing immigrant communities.

Given these realities and the significant distances from employment opportunities in downtown Toronto, many suburban residents have few options but to participate in unsustainable energy practices, such as spending hours commuting on highways and living in low density, less energy efficient housing developments. Low income, marginalized residents, who have far fewer opportunities to “vote with their feet”, are disproportionately affected by unregulated growth in real estate markets and urban sprawl and have fewer resources to practice NIMBYism (not-in-my-back-yard syndrome) of their own.

Even with the push toward privatization under neo-liberal regimes, as described above, provincial and municipal governments have taken a leadership role through the development and implementation of environmental and energy policy to develop local renewable energy resources for Toronto. However, energy policy in Toronto and initiatives to reduce Toronto’s carbon footprint are complicated by the Ontario provincial government’s and the Toronto municipal government’s overlapping jurisdictions relating to the governance over urban planning, transportation, and natural resources. In the discussion that follows, two provincial Ontario energy policies, The *Green Energy Act 2009* [31] and the *Places to Grow* policy [38] in 2004, both administered by the Ontario Ministry of Energy and Infrastructure Development are analyzed using an environmental justice lens to, first, critique the neo-liberal and green capitalism ideologies embedded in the policies. Second, we use environmental justice as a framework to examine the extent to which the green economy/green jobs and smart growth, as two energy solutions emerging from the *Green Energy Act* and *Places to Grow* policies, can concurrently address Toronto’s energy contradiction in light of the challenges of socially, economically, and spatially integrating immigrants. Given that the *Green Energy Act* 2009 is a relatively new policy, much of this analysis is based on a review of government and environmental non-governmental organizations’ documents and online reporting. In short, this paper seeks to contribute to both the void in the critical scholarship relating to Ontario’s *Green Energy Act* and to providing evidence of the need to bring environmental justice to the study of these policies.

### a. The *Green Energy Act* and Green Jobs

Established in May of 2009, the Ontario *Green Energy and Green Economy Act* is a significant policy tool for government support for renewable energy projects. In an effort to establish Ontario as a leader in the green economy, the *Green Energy Act* attempts to stimulate employment, or green jobs, and to fight climate change by encouraging the move away from coal-fuelled energy plants and toward using biomass, biogas, solar, and wind energy. In this regard, the *Green Energy Act* (“*GEA”*) outlines six specific investment areas: conservation and demand management; hydroelectric power; on-shore wind; bio-energy; waste energy recycling; and solar power [31]. Consistent with green capitalist thinking, advances in energy-oriented technology are seen as the means to resolve the present imbalance between energy demand and energy supply that characterized Toronto’s energy problems, in addition to new energy technology providing for local boosts to the Toronto economy through jobs and an improved global status for Toronto and the province of Ontario as leaders in the green economy. Embracing the green economy is positioned as a “win-win” situation for all stakeholders, including businesses and environmentalists, who are often seen as having incompatible objectives.

Core components of the *GEA* [39] include the goals to:

○ grant priority to purchase from green energy sources;
○ introduce a feed-in tariff (FIT) program as a mechanism to ensure the equal participation of the community energy sector and to provide for a reasonable rate of return on investment;
○ create an obligation for utilities to provide priority to green energy projects;
○ encourage the participation of First Nations and Metis as developers and owners in green projects;
○ invest $25 million to create the Community Power Corporation to assist local communities to develop viable projects; and
○ give priority to vulnerable consumers to ease their energy burden.

We believe that an environmental justice approach to energy policy would advocate for more active participation of affected communities as well as the questioning of relations of power implicated in and reproduce through the move toward green power. To its credit, the *Green Energy Act* of Ontario [31], acknowledges and seeks to address the needs of diverse stakeholders through, for example, seeking to work with First Nations and Metis in planning and implementing green energy projects; an emphasis on community power and the plans to establish a Community Power Corporation, and the acknowledgement of lower income people and their ability to pay as energy consumers. However, we believe that environmental justice takes us further, as an analytical approach, to examine the political interests of various actors. In this regard, we focus on four issues: the feed in tariffs program (or FIT) as a market driven approach; the political and economic reality of community power; the politics of including First Nations communities; and the promise of green jobs. Given that the implementation of the *GEA* and its programs are still in their nascant stages and there is, presently, a short record of outcomes, our comments are informed by some of the benefits and challenges raised in the grey literature on Ontario’s *GEA*.

On October 1, 2009, a feed-in tariff or FIT program for renewable energy was launched by the Ontario Ministry of Energy and Infrastructure as an essential component of the *Green Energy Ac*t. The FIT program is implemented by the Ontario Power Authority, which manages Ontario’s electricity grid and which receives directives from the Ministry. The FIT’s function as financial incentives for all players, including community power and First Nations stakeholders, is to produce electricity from green energy by offering stable guaranteed pricing for long term contracts [40]. In addition, “[T]he FIT program includes an incentive for community power in the form of a price adder of up to 1 cent extra per kWh and decreased security deposits” ([41], par. 17). The ultimate goal of the FIT programs is to contribute toward a phase out of coal-fired electricity by 2014.

Ontario’s FIT program in many respects mirrors the Renewable Energy Feed-in Tariff (REFIT) that operates in Germany. Both the Ontario and German programs involve state ‘command and control’ for pricing settings and guarantees [42]. Toke and Lauber’s ([42], p. 683) work, which analyzes the financing of renewable energy in both the UK and Germany, argues that, in Germany, even with the government’s involvement in regulating the pricing, REFITs still rely on elements of neo-liberalism because they foster competition in the selling of their products to developers, who will be inclined to keep their costs down. In the case of Ontario’s FIT program, competition will be generated by Ontario Power Authority that will control the increased demand from various new energy producers for contracts that allow for access to the grid. This is a significant change from the situation prior to the introduction of the *Green Energy Act*, when there were few players accessing the grid. Access to the power grid will, potentially, become the site where attempts to democratize participation in keeping with the goals of environmental justice will be the most challenging. As an example, on March 10, 2010, the Ontario Power Authority announced that 510 new renewable energy projects received contracts under Ontario’s new feed-in tariff incentive program. Under the FIT program, ninety percent of the contracts received were to corporations and large stakeholders for rooftop solar projects with only a small percentage of contracts going to small scale producers of alternative forms of renewable energy, such as water power projects or biogas projects [43]. In this regard, Deb Doncaster, Executive Director of the ENGO Community Power Fund, states, ‘

While we are overjoyed that the FIT is off to a good start, there are rules that inadvertently put community owned projects at a disadvantage and, as a result, only a handful are being announced here today. … One example of this is the one property-one contract rule, which restricts the number of projects on community college and university campuses and large municipal properties with multiple buildings, such as Exhibition Place in Toronto, important sites for community owned projects of varying scales ([43], par. 6).

Similarly, the Ontario Sustainable Energy Association, an advocacy non-governmental organization for the community power sector, in a letter dated October 15, 2009, addressed to the Minister of Energy of Infrastructure, draws attention to the FIT program’s bias toward commercial developers due to the challenge that community power groups were encountering in attempts to adhere to rules for eligible FIT contracts, such as the definition of participating landowners [44].

And yet, much of language in the *GEA* is intended to encourage and support community power. “Community Power is a class of sustainable energy projects that are owned, developed and controlled in full or in part (50 per cent or more) by residents of the community in which the project is located” [45]. Under this definition, community power involves homeowners, farmers, First Nations and Metis communities, cooperatives and municipalities as a diverse sector with varying capacities to become producers of green energy. The Community Power sector, as envisioned by the Ontario government and ENGOs (Ontario Sustainable Energy Association and its funding arm the Community Power Fund), draws on the community power experiences in Germany and Denmark. Feed in tariffs program (FIT) and MicroFIT for smaller scale projects are the essential components that ensure that all players can potentially equally contribute to renewable energy production and to ensure that a variety of players are included in a decentralized energy grid. The Community Power Fund has two principle programs to assist those in the community power sector: the Community Energy Partnerships Program (CEPP) and Community Power Capital (CP Capital). Launched in May 10, 2010, the CEPP, provides grants to fund the development and regulatory phases of community energy projects. Similar to the financial supports available for First Nations communities, the community funding presumes that communities have the requisite resources to establish projects that would be eligible for tariffs to provide the return on investment, whereas in terms of access to specialized expertise, person-hours and liquid assets they are outclassed by more powerful corporate stakeholders.

The First Nations Energy Alliance is a network of about 24 First Nations established in 2007 to encourage sustainable energy as an economic development strategy and to assist First Nations and Metis people in meeting their own energy needs [46,47]. The Alliance is partially funded by the Ontario Power Authority, which is responsible for implementing the *GEA*’s tariffs program. Through the Ontario Power Authority, the Ontario government has undertaken consultations with the First Nations Energy Alliance regarding the obligation to be respectful of Aboriginal territory and to forge partnerships. While many First Nations are generating renewable energy, for those First Nations in the planning process, two main challenges already been identified and are significant from the perspective of the politics of participation. The first barrier is the lack of access to Ontario’s energy grid. As Michael Fox, a founding Director of the First Nations Energy Alliance states:

Renewable energy policies in many European jurisdictions empower and obligate the local utility to connect projects to the grid and to facilitate projects by building grid capacity where it is needed. First Nations in Ontario need a *Green Energy Act* that allows communities to develop projects by ensuring grid access and capacity ([48], par. 11).

Second, the Ontario Power Authority uses a criteria points system to evaluate and shortlist green energy providers that are eligible for tariffs and supports. These criteria include: “environmental assessment, zoning, equipment, resource availability, proponent team and financial assessment” ([49], par. 20. Few First Nations groups have been able to meet these criteria. In April 2010, new Ontario Ministry funds (under the *GEA* to be administered by the Ontario Power Authority) were allocated to assist applicants under the Aboriginal Energy Partnerships Program (AEPP). This program has project requirements for eligibility including agreements in place to sell or transmit electricity [50].

Our preliminary analysis of components of the GEA suggests that priorizing economic growth in the *GEA* will result in social and environmental inequities as businesses, developers, and investors who have the benefit of access to upfront startup capital, time, expertise, and the knowledge to navigate the bureaucracy are privileged. The growing number of environmental non-governmental organizations who are advocating for and assisting community power groups and First Nations communities with gaining access to funding and the professional assistance needed to qualify for FIT contracts will in part help to address some of the apparent inequalities.

The green economy is another important objective of Ontario’s *Green Energy Act.* In the context of declining jobs in the manufacturing sector, green jobs are seen as a means to simultaneously foster economic development and fight climate change. Interest in green jobs and in the green economy are forging new partnerships between manufacturing unions and environmental nongovernmental organizations. Blue Green Canada, for example, brings together the United Steelworkers and Environmental Defense to encourage all levels of government in Canada to invest in green jobs, emphasizing in particular jobs in manufacturing, construction, and trades. In this regard, Blue Green appears to be responding to deindustrialization and restructuring in the global economy [51]. In May 2010, Blue Green Canada released a report that criticized the federal government’s investment in green jobs as weak relative to most other developed nations. Blue Green argues that Canada’s poor investment in green jobs is connected to the federal government’s attempt to protect its economic interest in the Alberta tar sands [51].

In a study commissioned by public and private stakeholder organizations that support the *Green Energy Act* (the Green Energy Act Alliance, Blue Green Canada, and the World WildLife Fund), Pollin and Garrett-Peltier [52] used Ontario, Canada, and US data to estimate the number of jobs that will be created with Ontario’s *Green Energy Act*. They examined three areas of job creation: direct effects (e.g., energy conservation management); indirect effects (e.g., suppliers such as the steel industry); and, induced effects (employment generated through goods and services that people employed in the first two categories would purchase). The authors suggest that the *Green Energy Act* will create 90,000 jobs in Ontario over 10 years, and that the majority of these jobs will offer ‘decent pay’ of over $20 per hour ([52], p. 6). However, they acknowledge that a significant minority will be low paying jobs in the construction and farming industries. It is noteworthy that, on account of Canada’s immigration policies, many newcomers to Canada would be overqualified for such jobs and therefore not beneficiaries of this new green job creation.

One initiative that attempts to directly integrate Toronto’s immigrants into green jobs is a two year pilot project “Green Opportunities: Reducing Barriers and Discriminatory Approaches to Increase Newcomer Participation in Environmental Activities”. This project funded by the Department of Citizenship and Immigration Canada aims to connect new immigrants in Toronto and Southwestern Ontario to green sector employment. The project is implemented by FutureWatch Environment and Development Education Partners, a Toronto-based environmental nongovernmental organization. Consistent with an environmental justice framework, this initiative seeks to address systemic and social networking barriers that often limit immigrants and other racialized peoples’ participation in environmental activities and industries. Green Opportunities objectives include: to improve newcomer integration into Ontario; to enhance the employment prospects of foreign-trained professionals, to promote the viability of Ontario’s Green Economy and community environmental sectors, and to promote environmental sustainability [53]. The final goals of the project include establishing formal partnerships and the development of a ‘best practices’ manual for newcomer engagement with an applied antiracism analysis [53].

### b. *Places to Grow*, Inner City Re-Development, and Smart Growth

*Places to Grow: Better Choices, Better Futures* established in 2004 outlines the Ontario government’s strategy to manage population growth and economic expansion and reconcile these with environmental considerations [38]. As previously noted, much of the population growth in Toronto and surrounding areas is projected to result from new immigration. As a result, *Places to Grow* is not only an environmental, urban development driven policy, but it also responds to and informs the settlement patterns of Toronto’s diverse communities. As a policy that seeks to curb further suburban expansion, *Places to Grow* emphasizes land use intensification, the re-development of former industrial sites (brownsfields), and compact development. In particular, the policy is aimed at pockets along the Lake Ontario waterfront specifically designated as future population and economic growth growth areas within the ‘Golden Horseshoe’ area. These emphases can be read as promoting energy reduction and energy sustainability, as it makes use of existing infrastructure and stimulates residential development in locations that are already well suited to public transportation and work/living communities.

As a direct result of initiatives like *Places to Grow*, inner city redevelopment in Toronto has transformed the downtown. Young professionals and groups that a generation ago had contributed to the expansion of Toronto’s suburbs by moving into low density housing developments are the demographic that is now choosing to live in new condominiums, lofts, and townhouses in downtown Toronto [54]. This occurs in the context of frozen federal funding for subsidized housing since the 1990s and the increased privatization of the housing market. Brownfield sites close to the commercial and financial centre and close to the coveted Lake Ontario waterfront have become hot properties in Toronto. Gentrification is pitting developers, real estate agents, and potential middle class residents, against the subsistence needs of marginalized groups and marginalized land uses pushed out from these areas, and creating new forms of environmental inequality in the process.

*Places to Grow* encourages urban development that draws foreign investors and businesses. Teelucksingh [17] argues that growth oriented development in downtown Toronto is pushed at the expense of provincial and municipal governments giving adequate attention to the procedural rights of stakeholders, including the need for diversity in the housing market in terms of housing type and tenure. Similar to Holifield’s [29] analysis of ‘deep neoliberalism’ hidden in US urban renewal initiatives that coopted discourses of environmental justice and community empowerment, the social inequalities associated with urban development in Toronto directed toward the global economy are often repackaged with the more socially acceptable discourses of smart growth or sustainable development [17].

“Smart growth” refers to a critique of low density urban sprawl and to the need to preserve greenfields and agricultural lands, reduce automobile dependency, and make more efficient use of existing inner city infrastructure. Smart growth, while environmentally necessary, will not be socially sustainable if targeted growth and development further marginalize and exclude low-income populations and deepen racial segregation. Smart growth development in Toronto, as an outcome of policies like *Places to Grow*, has not provided urban redevelopments that are accessible to all of Toronto’s diverse and multicultural communities. .

“New urbanism” is widely touted as another potential solution to balancing diverse interests in urban development. New urbanism began in the late 1980s to early 1990s and advocates for the renovation of brownfields with the explicit purpose of creating mixed income and mixed land use communities [55], and creating less car-dependant development. In contrast to “smart growth” discourses, which share similar critiques of urban sprawl, the new urbanism movement tries to address the needs of all citizens, including lower income people While there has certainly been a move toward increased mixed use zoning in Toronto, the same cannot be said of land use that would attract a mix of housing tenures and services for low income and marginalized people. New urbanism’s focus on both mixed land use and mixed income aims to avoid the exclusionary outcomes of gentrification.

## 5. Conclusions and Implications for Practice

This paper contributes to the literature on environmentalism, environmental justice, and energy use and reduction in Canada by uncovering the extent to which the green capitalist approach has impacted how state initiatives address the energy needs of marginalized communities in Toronto. The paper also illustrates the utility of applying an environmental justice frame to energy-related problems so as to analyze stakeholders’ positions in the context of neo-liberalism and green capitalism.

From the standpoint of contributing to new types of energy policies, an environmental justice approach includes a commitment to democratic and engaged community organizing at the grassroots level where marginalized communities are not simply unevenly subject to environmental justice problems, but are also central agents of change in their communities and strong, resourceful potential contributors to the common good. An environmental justice approach would advocate for the more active participation of affected communities, including immigrant communities, and furthermore (as illustrated in our review of the *Green Energy Act*) make the conditions for participation viable for marginalized communities to participate meaningfully in the community power sector and Ontario’s renewable energy grid.

We have also argued that environmental justice, as an alternative to green capitalism, can inform a more sustainable energy policy in Toronto. In our view, it can do so in the following ways:

○ By emphasizing the importance of broad-based networks that include, for instance, members of labour unions, immigrants and ethno-cultural groups, health care agencies, and educational institutions. The “Green Opportunities” project described above is a good example of a state funded initiative that is directed by a local ENGO.
○ by acknowledging that environmentalism in Canada has historically been alienating and exclusionary; constructing immigrants and lower income people as outsiders to environmental change. Forms of social exclusion and discrimination are barriers to immigrants and other marginalized groups more actively participating in energy programs. In some cases, applying an environmental justice perspective will mean reframing energy projects to have a greater focus on community needs.
○ by Recognizing that urban renewal is not advantageous if it deepens existing social and spatial inequities. Initiatives, such as increased funding for public transit, are not beneficial to all Torontonians, unless transit improvements are also made in suburban regions.
○ by Perceiving marginalized stakeholders, including low income residents, and racialized new immigrant communities, as not simply potential energy consumers, but also as active agents of change in their local communities where “green wealth” is shared equitably [56].
